# Idiopathic Orofacial Granulomatosis with Varied Clinical Presentation

**DOI:** 10.1155/2013/701749

**Published:** 2013-09-12

**Authors:** Rathy Ravindran, Anila Karunakaran

**Affiliations:** ^1^Department of Oral and Maxillofacial Pathology, Azeezia College of Dental Science & Research, Diamond Hills, Meeyannoor, Kollam-691537, Kerala State, India; ^2^Department of Oral and Maxillofacial Pathology, Kannur Dental College, Anjarakandy, Mamba, Kannur-670611, Kerala State, India

## Abstract

Orofacial granulomatosis is a granulomatous disease of orofacial region, which can occur for a variety of reasons. The clinical features are highly variable and sometimes so insidious that signs and symptoms are not frequently severe to cause alarm. The lips are most commonly involved with persistent/recurrent swelling. The medical history is very important as Crohn's disease and sarcoidosis can present oral manifestation. Other causes like mycobacterial infection, foreign body reaction, fungal infection, and allergy were excluded with further investigation to establish diagnosis. Here and we report a case of orofacial granulomatosis with a review of the literature.

## 1. Introduction

Orofacial granulomatosis (OFG) is a chronic inflammatory disorder characterized by persistent or recurrent soft tissue enlargement, oral ulceration, and a variety of orofacial features. The term orofacial granulomatosis was proposed by Wiesenfield et al. in 1985. Focal granulomas may occur anywhere in the oral mucosa or in the subcutaneous tissue of the skin where they present as localized firm mass that are occasionally multinodular. When diagnosis of noncaseating granuloma is made microscopically, the patient should be evaluated for several systemic diseases such as crohn's disease, sarcoidosis, TB and the local processes (Table 1) that may be responsible for similar oral lesion has to be ruled out. Orofacial granulomatosis is a term which encompasses variety of clinical presentation that upon biopsy reveal presence of nonspecific granulomatosis inflammation. Here we report a case of idiopathic orofacial granuloma and the etiology, diagnostic approach and treatment of orofacial granulomatosis reviewed.

## 2. Case Report

**Figure 1:**
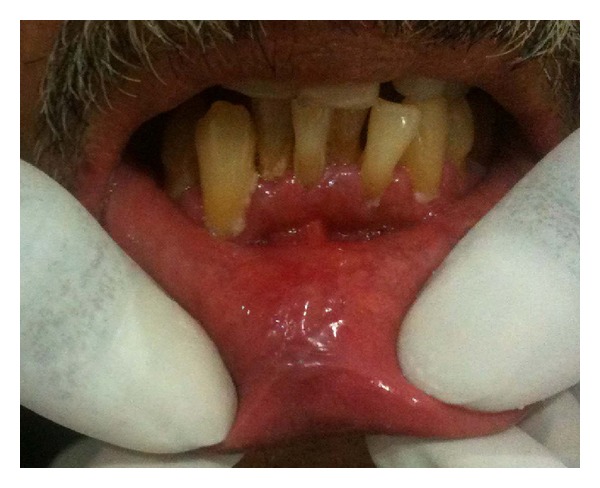
Diffuse swelling of lower lip.

**Figure 2:**
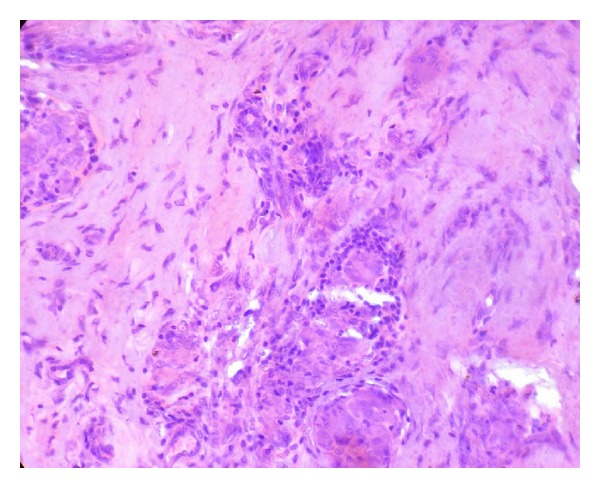
Aggregates of noncaseating granulomas (H&E 10x).

**Figure 3:**
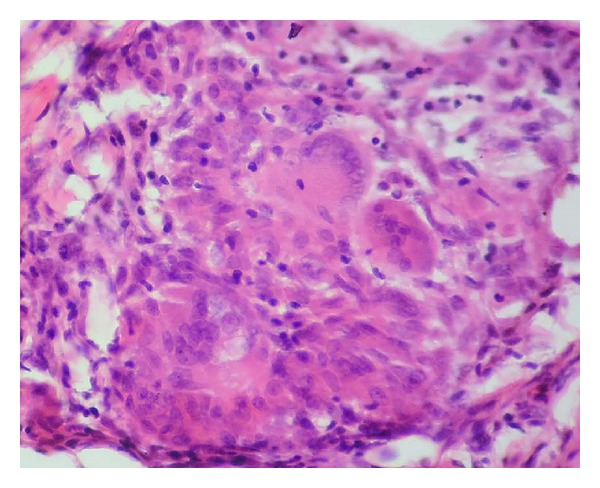
Granuloma showing peripheral lymphocytes, central histiocytes, and giant cell (H&E 40x).

**Figure 4:**
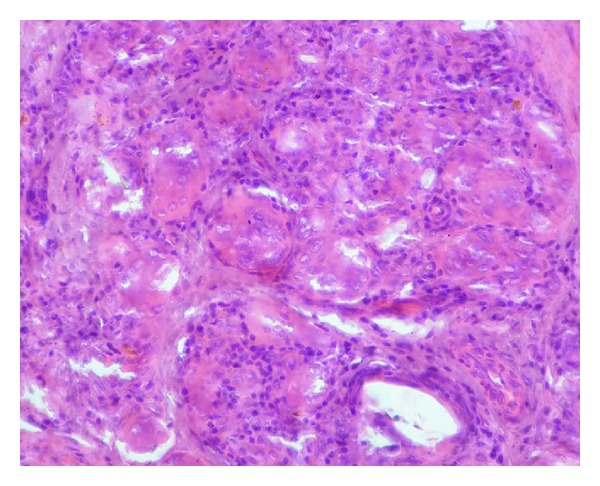
Granuloma with many multinucleated giant cells (H&E 40x).

A 40-year-old male patient reported to our OPD with complaint of swelling of lower lip. The patient gave a history of similar swelling 8 months back which was biopsied at another hospital and the report is unavailable. The medical history was noncontributory. Intraoral examination revealed diffuse swelling of lower lip (Figure 1). On palpation the swelling was nodular with diffuse margin, soft to firm in consistency. A provisional diagnosis of mucocele was made. Routine hematological examination was performed and all were with normal limits. Biopsy of the lip lesion revealed circumscribed aggregates of noncaseating granulomatous inflammation consisting of lymphocyte and epithelioid histiocytes with multinucleated giant cells. The granuloma consists of central aggregates of histiocytes with peripheral rim of inflammatory cells chiefly lymphocytes (Figures 2 and 3). The fibrous connective tissue showed areas of vascularity, multiple granulomas containing lot of giant cells suggestive of granulomatous lesion (Figure 4). Following this, patient was thoroughly investigated to rule out the list of granulomatous disease. Hematological investigation was performed for RBC count, differential count, platelet count, Hb%, ESR, serum folate, iron, and serum ACE (Angiotensin converting enzyme) levels were found to be within normal limits. The Mantoux test was negative. The chest radiograph did not reveal any pathology. Periodic acid Schiff stain for fungal organism and acid fast bacilli stain for mycobacteria were negative. No foreign material was detected. The patient gave no history of allergy. As the patient had normal hematological value and the medical history was not contributory for intestinal manifestation, further investigation for Crohn's disease was not undertaken.

 The above investigation ruled out tuberculosis, sarcoidosis, fungal infection, and Crohn's disease. The diagnosis of idiopathic orofacial granulomatosis was made. The patient reported with recurrent swelling. The patient was treated with biweekly intralesional injection of triamcinolone acetonide with simultaneous topical clobetasol propionate for 4 weeks. On review patient showed improvement. On followup for a period of one year the patient showed no recurrences.

## 3. Discussion

 The clinical and histopathological features of orofacial granulomatosis can be produced by a variety of underlying causes; this diagnosis is beginning of patient's evaluation [1, 2]. The conditions with granulomatous lesion histologically include Melkersson-Rosenthal syndrome, Crohn's disease, sarcoidosis, TB, hypersensitivity reaction, and angioneurotic edema [3] (Table 2).

In the present case the clinician's provisional diagnosis was mucocele and hence the lesion was submitted for histopathological examination. The clinical presentation of intraoral mucocele in some cases of sarcoidosis which led to its diagnosis has been reported [3].

 On histopathological diagnosis of noncaseating granulomatous inflammation, systemic evaluation was undertaken. The hematological evaluation and chest radiograph ruled out sarcoidosis and tuberculosis [4, 5]. Since the patients hematological investigation was within normal limits and did not give history of signs and symptoms of gastrointestinal discomfort further investigation of colonoscopy and biopsy is not required [2, 6]. Investigations were performed to exclude diseases with similar clinical and histopathological feature. Orofacial granulomatosis is a disease with wide spectrum of clinical presentation. The causative agent in the present case is unknown; hence it is categorized as idiopathic Orofacial granulomatosis.

 The exact cause of orofacial granulomatosis is still unknown, although several theories have been suggested including infection, genetic predisposition, and allergy [7]. The etiological agents such as food substances, food additives, dental material microbiological agents have been proposed, but its pathogenesis is uncertain. A delayed type of hypersensitivity reaction appears to play a role, but the antigen inducing immunological reaction varies in individuals. The evidence for role of genetic predisposition to disease is sparse. Tilakaratne et al. propose the term idiopathic orofacial granulomatosis as better term for cases restricted to oral region without any identifiable known granulomatous disease and diagnosis should not be changed until patient develops systemic manifestation of specific granulomatous condition [8]. The various treatment modalities include intralesional injection, topical and systemic steroids, and surgical excision [9].

## 4. Conclusion

 Orofacial granulomatosis can be a distinct clinical disorder or can be an initial presentation of underlying systemic disease such as crohn's disease or sarcoidosis. Hence an early diagnosis of orofacial granulomatosis can identify an underlying disease as cases have been reported which had initial manifestation of orofacial granulomatosis. Hence followup for the patient is advised as a step in prevention of a possible systemic granulomatous disease.

## Figures and Tables

**Table 1:** Oral granuloma.

Systemic causes	Local causes
Chronic granulomatous disease	Chronic oral infection
Crohn's disease	Foreign material
Sarcoidosis	Allergy
Tuberculosis	

**Table 2:** Differential diagnosis of orofacial granulomatosis.

Disease	Features different to OFG
Crohn's disease	Have illeal and/or rectal disease
Sarcoidosis	Pulmonary, cutaneuos, lacrimal, salivary, neurological, skeletal features
Allergic angioedema	Nonpitting oedema of lips, tongue, pharynx, face. History of atopic disease
Miescher's cheilitis	Labial enlargement, similar histopathology to OFG
Melkerson-Rosenthal syndrome	Labial enlargement, fissuring of tongue, facial nerve palsy variant of OFG
Cheilitis glandularis	Labial enlargement with ulcers. Mild acute and chronic inflammation (without granuloma) within minor salivary glands of lip
Tuberculosis	Rarely lips. Usually contain caseating granuloma
